# Visual perception and processing in children with 22q11.2 deletion syndrome: associations with social cognition measures of face identity and emotion recognition

**DOI:** 10.1186/s11689-016-9164-7

**Published:** 2016-08-17

**Authors:** Kathryn L. McCabe, Stuart Marlin, Gavin Cooper, Robin Morris, Ulrich Schall, Declan G. Murphy, Kieran C. Murphy, Linda E. Campbell

**Affiliations:** 1 Priority Research Centre for Brain and Mental Health Research, The University of Newcastle, Callaghan, Australia; 2 Department of Psychiatry and Behavioral Sciences, University of California, Davis, CA USA; 3 Schizophrenia Research Institute, Sydney, Australia; 4 School of Psychology, University of Newcastle, Callaghan, Australia; 5 King’s College Institute of Psychiatry, Psychology and Neuroscience, London, UK; 6 Department of Psychiatry, Royal College of Surgeons in Ireland, Dublin, Ireland; 7 Hunter Medical Research Institute, Newcastle, Australia; 8 Priority Research Centre GrowUpWell, The University of Newcastle, Callaghan, Australia; 9 School of Psychology, University of Newcastle, Science Offices, Ourimbah, NSW 2258 Australia

**Keywords:** 22q11.2 deletion syndrome (22q11DS), Perceptual organisation, Visual integration, Object recognition, Face processing, Social cognition

## Abstract

**Background:**

People with 22q11.2 deletion syndrome (22q11DS) have difficulty processing social information including facial identity and emotion processing. However, difficulties with visual and attentional processes may play a role in difficulties observed with these social cognitive skills.

**Methods:**

A cross-sectional study investigated visual perception and processing as well as facial processing abilities in a group of 49 children and adolescents with 22q11DS and 30 age and socio-economic status-matched healthy sibling controls using the Birmingham Object Recognition Battery and face processing sub-tests from the MRC face processing skills battery.

**Results:**

The 22q11DS group demonstrated poorer performance on all measures of visual perception and processing, with greatest impairment on perceptual processes relating to form perception as well as object recognition and memory. In addition, form perception was found to make a significant and unique contribution to higher order social-perceptual processing (face identity) in the 22q11DS group.

**Conclusions:**

The findings indicate evidence for impaired visual perception and processing capabilities in 22q11DS. In turn, these were found to influence cognitive skills needed for social processes such as facial identity recognition in the children with 22q11DS.

## Background

22q11.2 deletion syndrome (22q11DS) is a genetically determined microdeletion syndrome with a complex medical phenotype that includes cardiac and palatal anomalies [1]. In addition, mild intellectual and developmental delays affect almost all children born with the syndrome as well as specific cognitive impairment. As children with the syndrome grow up, they are at an increased risk of neurodevelopmental problems such as anxiety and autism spectrum disorder (ASD) [2–4]. Difficulty processing social information such as recognising facial identity and facial expressions of emotion as well as understanding the intentions of others is also common in 22q11DS [2, 3]. In addition, the 22q11.2 deletion is associated with marked impairment of visuo-spatial abilities [4, 5]. However, visual perception and processing ability has not been comprehensively explored in this group and it is not clear what effect impaired visual perception and processing may have on social-perceptual processes such as facial identity and facial emotion recognition in 22q11DS.

Visual perception and processing (also known as perceptual organisation or visual integration) refers to the transfer of local visual neuronal output into complex “global” structures [6] and includes the techniques used to gather visual information from the environment and integrate these with other senses (bottom-up processing) as well as accessing and incorporating previously integrated information from experience, motivation and development (top-down processing). These processes help us derive understanding and meaning from our perceptual experiences and in turn guide our behaviour [6]. Experimental tasks of visual perception are used to assess the different features of visual perception and processing that contribute to object recognition capability. These include basic perceptual tasks such as figure-ground segmentation and same/different matching of visual features such as orientation, length or size as well as asking participants to match objects that are displayed from different viewpoints. In addition, the ability to access stored perceptual knowledge about an object, such as object name, or to match items on the basis of function or some other associative criteria, are examined by tasks of visual perception and processing.

Despite behavioural evidence indicating selective impairment of visual-spatial memory [4], impaired sensory processing [7, 8] and lower scores on measures of non-verbal IQ than verbal IQ in 22q11DS, few studies have examined aspects of visual perception and processing such as object recognition. When examined more closely, there is good evidence to suggest that people with 22q11DS have difficulties with visual perception and processing. For example visual perception and visual-motor integration difficulties have been reported in 22q11DS children as young as 5 years old [9]. In this study and others, the 22q11DS group performed at least one standard deviation below the mean on an object-matching task [9, 10]. Adults with 22q11DS also show visual perception and processing difficulties with impaired performance on two of four object recognition subscales (silhouette and shape decision) of the visual object and space perception battery (VOSP) [11]. In addition, 22q11DS participants show evidence of a selective impairment discriminating local details when they are contained within global forms [12] indicating difficulties with both visual-spatial information processing and form perception. Work reported previously by our group explored visual-spatial information processing in adolescents with 22q11DS and identified impairments in their ability to discriminate between different types of facial displays of emotion [3] as well as different types of complex scenes [13] compared to age and gender matched typically developing peers. Here, eye-tracking measurement indicated altered visual information processing strategies when viewing face and non-face stimuli. In addition, children with 22q11DS display impaired performance on the social-perceptual aspects of the MCR face processing battery [2]. These studies indicate that some people with 22q11DS have difficulty processing facial information as well as complex scenes, especially when images are presented from a non-typical perspective. Taken together, findings suggest that dysfunction in visual perception and processing of complex stimuli are likely to contribute to the social cognitive and social competence problems observed in people with 22q11DS.

The study presented here investigated visual perception and processing performance of children with 22q11DS and an age and socio-economic status matched sample of typically developing sibling controls (HC) using behavioural measures of object recognition. It also examined whether components of visual perception and processing are associated with social-perceptual components of face processing. On the basis of existing literature, it was hypothesised that (1) the 22q11DS group would show poorer performance on measures of visual perception and processing ability and that (2) visual perception and processing would predict performance on social-perceptual measures of face and identity recognition.

## Method

### Participants

Included in the study were 49 children with genetically confirmed 22q11DS (28 female, mean age 11 years, SD ±2.9, mean IQ 66.3, SD ±8.6) and 30 non-22q11DS sibling controls (13 female, mean age 11 years, SD ±2.5, mean IQ 103, SD ±13). Deletion of chromosome 22q11 was confirmed using fluorescence in situ hybridisation (FISH). No significant difference in mean age (*t* = 0.09, *p* = 0.93) or gender distribution (*χ*^2^ (1) = 1.42, *p* = 0.23) was present between the groups.

Exclusion criteria included the presence of the clinical phenotype of 22q11DS but the absence of a 3 Mb 22q11.2 deletion for the clinical group as well as the presence of a clinically detectable medical disorder known to affect brain function (e.g. epilepsy or hypertension), a history of head injury and moderate/severe intellectual impairment (WISC IQ <50) for both the 22q11DS and control groups. Additional exclusion criteria for control participants included the presence of a current or past diagnosis of an axis I psychiatric disorder. The absence of a 22q11 chromosome deletion was confirmed in all sibling controls. One child with 22q11DS was excluded due to moderate intellectual impairment. Prior to testing, consent was obtained from the participants or, if aged 15 years or younger, from parents or guardians. The study was approved by the local ethics committee at the Institute of Psychiatry, South London and Maudsley Trust (067/00). The demographic characteristics of the final sample are presented in Table 1.

**Table 1:** Participant characteristics

	22q11DS Mean (SD)	HC Mean (SD)
Age	11.0 (2.9)	11.1 (2.5)
Gender (% female)	57 (*n* = 28)	43 (*n* = 13)
FIQ	66.33 (8.6)	103.87 (13.0)
VIQ	71.08 (10.76)	105.4 (14.75)
PIQ	66.69 (10.43)	100.8 (10.29)

*FIQ* full-scale intelligence quotient, *VIQ* verbal intelligence quotient, *PIQ* performance intelligence quotient

### Stimuli and tasks

#### Intellectual functioning

General intellectual functioning was assessed using the Wechsler Intelligence Scale for Children-III (WISC-III) [14].

#### Object recognition: Birmingham Object Recognition Battery

Visual perception and processing was measured using sub-tests of the Birmingham Object Recognition Battery (BORB; 15). The BORB sub-tests used in the present study assessed two components of visual perception and processing: (i) recognition of visual objects across different viewpoints (field dependence/independence, form perception) and (ii) the ability to access stored knowledge of visual stimuli (object recognition; implicit memory and semantic memory). Form perception was examined using Foreshortened Match and Minimal Features Match sub-tests. In these sub-tests, the participant was required to match a picture of an object taken from a standard viewpoint with a picture of the same object taken from another viewpoint, a third picture of a different object providing a distracter item. For the minimal features sub-test, the main identifying feature of the object was concealed, while in the foreshortened match sub-test, the main feature was maintained but presented from an unusual viewpoint. The form perception items were separated into easy and hard sub-tests. On the form perception tasks, participants were presented with an object target stimulus at the top of the page and response option object stimuli at the bottom of the page. The tasks require participants to select a stimulus matching the target either based on local (minimal features) or global (foreshortened view) processing strategies. These tasks assessed “pre-categorical” processing of visual stimuli [15]. Object recognition was tested via the picture naming sub-test, implicit memory was examined via the object decision sub-test and semantic memory via the associative matching sub-test. On these sub-tests, participants were presented with stimuli and asked to either provide information about or associated with an object or to name the object (e.g. animal). In addition, the figure-ground segmentation sub-test was included to assess how well the participant was able to group different components of an object together, while discriminating them from surrounding objects. Reaction time to naming pairs or triplets of objects was recorded when stimuli were either overlapping or not overlapping. Object recognition was calculated by a ratio of overlapping: not overlapping, this value is expected to be equal to or approaching 1:1 for participants with normal visual processing. All tasks were administered consistent with instructions described by Riddoch and Humphreys [15].

#### The Embedded Figures Test

The Embedded Figures Test (EFT) [16] was used to assess the ability of participants to extract information from a surrounding gestalt (i.e. field independence). In this task, participants had to identify a shape embedded in a more complex shape.

#### Face and emotion processing

Face processing was assessed using that MRC Face Processing Skills Battery, a procedure suitable for testing children [17]. The specific face processing tasks used in the present study have been reported in detail previously [2]. Briefly, the battery examined four aspects of face processing: identity, emotion, eye gaze and facial speech (sound). There were a total of 14 tests in the battery, and stimuli were presented on a grey background and were approximately 5.5 × 4 cm in size and presented to participants on an A4 size paper. Each of the sub-tests increased in difficultly over the trial. The Identity test contained five sub-tests with 16 trials each. Here, the participants were required to determined which face from the two presented before them belonged to the same child as a face presented above. The response stimuli differed in terms of age, gender, or appearance. In other sub-tests, the stimuli were the same individual but a key feature was removed (e.g. hair, ears) or a feature was obscured (e.g. grey circles over the eyes). The emotion task consisted of three sub-tests consisting of 12 trials each. Participants were required to provide a verbal label to emotion stimuli (happy, sad, angry or surprised) or they were instructed to point to the matching emotion from pairs of faces (e.g. ‘which is happy?’) or which of the two faces ‘feel the same’ as a target stimulus.

### Statistical analyses

Statistical analysis was carried out using SPSS (Version 22.0 IBM Corp. Armonk, NY). Between-group differences in age were assessed using independent samples *t* test and *X*^2^ test for categorical variables (gender distribution) with *p* < 0.05, two-tailed. IQ differences were not controlled for statistically in the main analysis as there was a greater than two standard deviation difference between the HC and 22q11DS groups thus IQ was treated as a group defining characteristic.

Data distribution was examined and Kolmogorov-Smirnov values indicated that several variables were non-normally distributed; therefore, non-parametric Mann-Whitney *U* tests were performed to explore pattern of performance between groups. Multiple regression analysis was used to model the contribution of visual perception and processing and age to performance on social-perceptual measures of face processing (facial emotion and facial identity recognition). It should be noted that the number of participants differs slightly between analyses therefore the sample size is reported for the separate analyses. Bonferroni adjusted significance level was set to between *p* = 0.01 and 0.03 to control for the increased possibility of type I error due to the number of tests conducted.

## Results

### Visual perception and processing

#### Object recognition (Birmingham Object Recognition Task)

Figure-ground segmentation sub-test performance showed no group differences once adjusted for multiple comparisons (single: *U* = 372, *N* = 66, *p* = 0.04, *r* = 0.23; double: *U* = 523, *N* = 66, *p* = 0.49, *r* = 0.08; triplet *U* = 374, *N* = 66, *p* = 0.03, *r* = 0.23). Participants with 22q11DS had significantly lower scores (poorer performance) on BORB basic perception sub-tests of form perception (easy: *U* = 367, *N* = 79, *p* < 0.001, *r* = 0.43; hard: *U* = 326.5, *N* = 75, *p* < 0.001, *r* = 0.43). The 22q11DS group also showed poorer performance on visual object recognition (*U* = 409, *N* = 78, *p* = 0.001, *r* = 0.37), implicit (*U* = 426, *N* = 77, *p* = 0.003, *r* = 0.33) and semantic memory (*U* = 353, *N* = 78, *p* < 0.001, *r* = 0.44) (Bonferroni adjusted significance level *p* = 0.01). See Table 2 for mean (SD), median and statistical tests of significance.

**Table 2:** Visual perception and processing performance in 22q11DS and HC participants

	22q11DS Mean (SD)	Median	HC Mean (SD)	Median	Statistic (*p*), effect size (*r*)
BORB					
Field dependence/independence					
Figure-ground segmentation					
Single letters	0.90 (0.1)	0.92	0.84 (0.1)	0.84	0.04 (0.23) NS
Paired drawings	0.97 (0.2)	0.97	0.88 (0.1)	0.89	0.40 (0.08) NS
Triplet letters	0.96 (0.1)	0.95	0.95 (0.2)	0.88	0.03 (0.23) NS
Form perception					
Form perception easy sub-tests (easy foreshortened + easy minimal features)	47.20 (2.78)	48.0	49.13 (1.38)	50.0	0.001 (0.43)
Form perception hard sub-tests (hard foreshortened + hard minimal features)	38.91 (4.1)	40.0	41.76 (1.8)	43.0	0.001 (0.43)
Visual object recognition					
Picture naming	12.56 (1.87)	13.0	13.70 (1.66)	14.0	0.001 (0.37)
Implicit memory					
Object decision (easy)	28.04 (3.94)		29.83 (1.49)		
Object decision (hard)	24.7 0(3.48)		26.53 (3.03)		
Implicit memory sub-total	53.04 (5.88)	54.0	56.37 (3.36)	57.0	0.003 (0.33)
Semantic memory					
Associative matching	27.29 (3.07)	28.0	29.27 (1.08)	30.0	0.001 (0.44)
Embedded figures test					
Total score	21.10 (4.0)	22.0	22.22 (1.85)	23.0	0.5 (0.09) NS
Reaction time (s)	33.89 (17.1)	30.3	26.76 (12.06)	24.3	0.07 (0.22) NS

*NS* not significant after correcting for multiple comparisons

#### Field dependence/independence (Embedded Figures Task)

Performance on accuracy and reaction time on the embedded figures task did not differ between the groups (accuracy: *U* = 488, *N* = 68, *p* = 0.5, *r* = 0.09; reaction time: *U* = 404, *N* = 68, *p* = 0.07, *r* = 0.22, see Table 2).

#### Face emotion processing

As has been reported previously [2], the 22q11DS group displayed poorer performance on face processing compared to the HC group (Bonferroni adjusted significance level *p* = 0.01): face identity (*U* = 114, *N* = 79, *p* < 0.001, *r* = 0.71) and facial emotion (*U* = 202, *N* = 79, *p* < 0.001, *r* = 0.62) (see Table 3).

**Table 3:** Face processing performance

	22q11DS (*n* = 30)			HC (*n* = 49)			
	Mean (SD)	Median	Accuracy (%)	Mean (SD)	Median	Accuracy (%)	Statistics (*p*), effect size (*r*)
Face processing							
Identity (0–80)	39.71 (8.43)	39.0	49.64	62.83 (14.12)	72.0	78.54	*p* < 0.001 (0.71)
Emotion (0–36)	16.57 (6.57)	20.0	46.03	22.4 (3.73)	23.0	62.22	*p* < 0.001 (0.62)

*NS* not significant after correcting for multiple comparisons

### Visual perception and processing: associations with demographic characteristics

IQ-independent impairment in visual perception has been reported previously [10, 18], though not consistently [9] in the 22q11DS literature. Therefore, we first explored associations between IQ and visual perception and processing with tasks on which performance between the groups did not differ (i.e. embedded figures, figure-ground segmentation). No association between IQ and task performance (*p*’s >0.05) was demonstrated on these measures. Therefore, IQ was excluded from subsequent multiple linear regression models. Age but not gender correlated with performance on visual perception and processing in the 22q11DS group. Age correlated positively with form perception (easy: *r* = 0.46, *n* = 49, *p* = 0.001; hard: *r* = 0.29, *n* = 46, *p* = 0.006) as well as semantic memory (*r* = 0.43, *n* = 48, *p* = 0.002). For the HC group, age was positively correlated with form perception (hard: *r* = 0.39, *n* = 29, *p* = 0.04; easy: *r* = 0.45, *n* = 30, *p* = 0.01), object recognition (*r* = 0.38, *n* = 30, *p* = 0.04) and implicit memory (*r* = 0.54, *n* = 30, *p* = 0.002).

### Visual perception and processing predictors of social perception

Preliminary analyses were conducted to test for violations of the assumptions of normality and multicolinearity. The ability of visual perception and processing (form perception, visual object recognition, implicit memory, semantic memory and field dependence/independence) to predict performance on social-perceptual processing (face identity and emotion recognition) was tested together and separately for the 22q11DS and HC groups using multiple linear regression. Age was entered into the regression model first followed by the BORB sub-test items.

#### Facial identity

Multiple regression analysis was used to assess the ability of form perception, visual object recognition, implicit memory, semantic memory and age to predict performance on facial identity recognition across groups. The total variance explained by the model was 30.6 %, (*F*(6, 73) = 6.38, *p* < 0.0001). Form perception (easy) (beta = 0.31, *p* < 0.03) made the strongest significant contribution to explaining performance of facial identity performance, followed by form perception (hard) (beta = 0.26, *p* = 0.05) the remaining predictor variables (age, gender, semantic memory, implicit memory and object recognition) failed to contribute significantly to the model (*p*’s >0.05). When examined separately by group, the model failed to significantly predict performance on facial identity in the HC group. In contrast, for the 22q11DS group, the total variance explained by the model increased 34.6 %, (*F*(6, 44) = 4.88, *p* < 0.001) with form perception (hard (beta = 0.40, *p* = 0.02) again making the strongest significant contribution. Similar to the combined model, the remaining predictor variables (age, gender, form perception (easy), semantic memory, implicit memory and object recognition) failed to contribute significantly to the model, however also included was form perception (easy) (*p*’s >0.05).

#### Facial emotion

The same variables (form perception, visual object recognition, implicit memory and semantic memory and age) were then used to predict performance on facial emotion recognition. The regression model did not predict facial emotion performance in the 22q11DS or HC groups or when the groups were combined.

## Discussion

The present study sought to characterise visual perception and processing ability in children with 22q11DS compared to a HC group and to determine whether components of visual perception and processing predicted the ability of participants to perform the social-perceptual processes of facial identity or facial emotion recognition. We report significant impairment of visual perception and processing among children with 22q11DS compared to HC participants. The 22q11DS group demonstrated poorer performance on all measures of visual perception and processing, with greatest impairment on processes relating to form perception as well as object recognition and semantic memory. Participants with 22q11DS also demonstrated poorer performance on face emotion and facial identity recognition compared to the TD group. Visual perception and processing was shown to make a significant and unique contribution to higher order social-perceptual processing in the 22q11DS group. Specifically, form perception predicted accuracy on a measure of facial identity recognition.

While participants with 22q11DS demonstrated poorer performance across all sub-tests of visual perception and processing compared to the HC group, they demonstrated particular difficulty identifying pictures when they were presented from unusual views or when crucial details were obscured. These form perception tasks assess “pre-categorical” processing of visual stimuli [15] and in the present study suggest that visual processing is impaired in 22q11DS at a pre-categorical level related to local and global processing abilities and that precedes the involvement of implicit and semantic memory. However, in addition to visual perceptual processes, the present study also demonstrated impaired implicit and semantic processes involved in object recognition in 22q11DS.

Our findings are consistent with previous studies that report specific neuropsychological impairment in visual perception and processing including visual-spatial memory [4], numerical ability [5], multiple object tracking [19], visual perception and visual integration [9]. These neuropsychological findings in 22q11DS are coupled with more recent psychophysiological and neuroanatomical evidence indicating altered visual processing [20] and the neural circuitry implicated in these processes compared to typically developing controls [21, 22]. Further, a visual processing study using event related potentials explored potential genetic influences in 22q11DS to visual processing and showed enhanced feed forward and reduced feedback activity on a texture segregation task [20]. The authors interpreted these findings as further evidence of abnormal recurrent visual processing indicative of impaired connectivity between higher and lower visual cortical areas in 22q11DS. Interestingly, recurrent visual processing activity was mediated by the interaction of COMT polymorphism and plasma proline levels, with high proline negatively impacting visual processing in the COMT (met) genotype subgroup.

It is not yet clear whether visual perceptual and processing impairment represents a specific manifestation of the 22q11DS phenotype. 22q11DS is associated with impairment of multiple processes that contribute to object perception. For instance, in Ellis and Young’s [23] model of object recognition, impairment is observed in 22q11DS at both the form perception stages *and* higher level stages that are reliant on memory functions involved with semantic processing and naming of objects. Several studies have attributed visual impairment to cardiac or neural crest development abnormalities in 22q11DS [4, 24]. It is also worth noting that ocular abnormalities are relatively common in 22q11DS. A comprehensive ophthalmological study conducted by Casteels et al. [24] on a sample of children with 22q11DS reported a variety of ocular impairments in the group including abnormal ocular vascular flow in 75 % of participants (i.e. tortuosity of the blood vessels). However, the authors noted overall normal visual acuity in the 22q11DS participants and attributed other ophthalmological abnormalities to neural crest development and congenital heart defects. They also noted that while abnormalities were likely to impact reading and learning ability, these impairments were responsive to intervention.

In partial support of our second hypothesis, a key finding of the present study was that visual perception and processing (form perception) made a significant and unique contribution in the 22q11DS group to predict performance on a social-perceptual measure of identity recognition. These findings indicate support for the contribution of visual perception and processing to higher order social cognitive processes. Interestingly, the same model of object recognition measures did not contribute significantly to emotion recognition performance. These findings run counter to our expectations, as we would expect similar recruitment of pre-categorical processes such as form perception for both face emotion and identity recognition. This unexpected finding may be explained by difference in the number of items on the social cognition tasks (face emotion = 36 items, face identity = 80 items). Fewer face emotion items may explain the smaller variance in participant’s emotion recognition task responses (variance = 307) than their identity recognition task responses (variance = 383).

It is interesting to note that participants with 22q11DS did not differ from the HC group in their ability to extract information from a surrounding gestalt. Performance on the EFT and to a lesser extent figure-ground segmentation (FGS) indicated that the 22q11DS group, while taking longer (though not statistically significantly), were able to accurately extract shapes from figures. Thus, we were unable to replicate previous 22q11DS findings showing a selective impairment extracting details from global forms [12]. This finding was unexpected given the visuo-attentional and visuo-spatial difficulties reported in 22q11DS. Differences in task design between studies may partially explain these findings. For instance, the EFT and FGS tasks require participants to identify simple shapes within more complex stimuli, whereas the task described by Giersch and colleagues [12] were of a more complex, multi-level design. The task features described by Giersch and colleagues that most closely related to the EFT, were the explicit processing of local and global information and the authors found that participants with 22q11DS had greater difficulty identifying differences between stimuli at both the local and global level and neither age or IQ explained these differences.

Recent meta-analysis of perceptual organisation in schizophrenia, for which 22q11DS is a risk factor, indicates impairment on EFT in schizophrenia high risk populations with a preference for global processing of stimuli [25]. However, elevated rates of autism spectrum disorders are also reported in 22q11DS [26, 27] which in turn, is associated with a preference for local processing and weak central coherence [28]. In addition, individuals with high autism or schizotypy traits demonstrate divergence local and global processing on the EFT [29]. Therefore, future studies of perceptual organisation in 22q11DS may benefit from closer examination of participants clinical profile in order to examine more closely the contribution of psychiatric symptomatology to task performance.

### Limitations

Limitations associated with the present study primarily relate to the selection of the control group. Firstly, as is common to studies of 22q11DS and other disorders characterised by intellectual disabilities, the HC group were not matched for IQ. It is possible that our findings reflect group differences in intellectual functioning as other studies have reported that IQ is associated with measures of visual perception and processing in young children (aged 5; [9]). Thus, a potential limitation of the study is the absence of an intellectually matched control group without the 22q11.2 deletion. However, this approach is not without its own limitations. First, other groups with intellectual impairment demonstrate face and emotion recognition abilities (e.g. William syndrome, [30]). Secondly, while groups may be matched on intellectual functioning, variable clinical phenotype of these groups may be problematic. For these reasons, we selected a sibling control group, who were both age and socio-economically matched to the 22q11DS group. Further, we ruled out the potential contribution of IQ by examining associations between measures of visual perception and processing tasks on which the groups did not differ in performance and we showed no association between IQ and EFT or FGS. Based on these finding we would argue that the visual perception and processing difficulties observed in 22q11DS are not explained by general intellectual impairment. And for these reasons, IQ was not included in subsequent regression models. An additional consideration and potential limitation related to the measures of face processing (emotion and identity tasks). Differences between these tasks may have limited our ability to explore patterns of association between these and visual perception and processing measures.

### Clinical implications

Visual perception and processing, like ocular abnormalities, impact learning, reading ability and other day-to-day functions. The findings from the current study indicate that impaired form perception may contribute to difficulties with higher order social cognitive processes in the same sensory modality. Continued examination will help determine if the 22q11DS phenotype extends to include fundamental visual perceptual impairment. 22q11DS is a risk factor for psychosis. Psychotic disorders, particularly diagnosis of schizophrenia is associated with impairment in visual perception and processing and social cognition. Moreover, visual perception and processing impairment is present in groups at clinical high risk for schizophrenia [31] and is shown to influence functional outcome in schizophrenia mediated by social perception [32]. Thus, it would be valuable in future 22q11DS studies to explore longitudinally whether visual perception and processing and face processing difficulties are predictive of later psychosis status. Second, visual perception and processing as well as social cognition respond to both targeted behavioural and cognitive remediation. Early identification and intervention of visual perception and processing difficulties could yield improvement in, for example, the domains of reading ability as well as social interaction and functioning.

## Conclusions

The present study provides further evidence for impaired visual perception and processing in 22q11DS and the association of these with social-perceptual processes such as identity recognition.

## Abbreviations

22q11DS, 22q11.2 deletion syndrome; ASD, autism spectrum disorder; BORB, Birmingham object recognition battery; EFT, embedded figures task; FGS, figure-ground segmentation; FIQ, full-scale IQ
